# Expression of pyroptosis-related genes are correlated with immune microenvironment and predict prognosis in ESCA

**DOI:** 10.1007/s00432-023-04958-x

**Published:** 2023-06-12

**Authors:** Youmeng Shi, Qiuxing Yang, Guomei Tai, Xudong Chen

**Affiliations:** 1 Department of Radiotherapy, Nantong Tumor Hospital and Nantong University, Nantong, China; 2 Cancer Research Center Nantong, Nantong Tumor Hospital and Affiliated Tumor Hospital of Nantong University, Nantong, China; 3 Department of Radiotherapy, Nantong Tumor Hospital and Affiliated Tumor Hospital of Nantong University, Nantong, China; 4 Department of Pathology, Nantong Tumor Hospital and Affiliated Tumor Hospital of Nantong University, Nantong, China

**Keywords:** Pyroptosis, Esophagus cance, Prognosis, Immune microenvironment

## Abstract

**Objectives:**

Pyroptosis-related genes (PRGs) are abnormally expressed in a variety of gastrointestinal tumors, this study aimed to investigate the role of pyroptosis genes in assessing the prognosis of esophageal cancer (ESCA).

**Methods:**

Through consensus clustering, we identified two subtypes associated with PRGs. After Lasso regression and multivariate Cox regression analysis, a polygenic signature based on six prognostic PRGS was constructed. Afterwards, we combined the risk score with clinical predictors to construct and validate a PRGs-associated ESCA prognostic model.

**Results:**

Through analysis, we Successfully constructed and validated a PRGs-associated ESCA prognostic model that predicts ESCA survival and correlates with the tumor immune microenvironment.

**Conclusion:**

Based on PRGs features, we established a new ESCA hierarchical model. This model has important clinical implications for ESCA patients, both in terms of assessing prognosis and in terms of targeted and immunotherapy.

## Introduction

Esophageal cancer (ESCA) is a global malignant tumor, ranking seventh and sixth in all tumor-related morbidity and mortality, respectively, with the highest incidence in men and women in East Asia (Hyuna et al. 2021). At the same time, ESCA has a low 5 year survival rate, which is only 20–30% (Zhang et al. 2022a, b). However, the risk of recurrence and metastasis is higher. There are many elements for the poorer prognostic outcome of ESCA, including occult initial symptoms, easy to metastasis, radiotherapy resistance and recurrence (Pennathur et al. 2013).

In the past few years, treatments such as multidisciplinary treatment and surgical treatment have developed rapidly and made some progress, but the median survival time of ESCA cases is still only 10 months (Wang et al. 2020). In addition, it is of great significance to reveal the characteristics of related prognostic genes in view of the limited prediction of prognosis in patients with ESCA.

Pyroptosis is called inflammatory necrosis of cells, which is a form of inflammation of programmed cell death. Studies have shown that pyroptosis is mediated by the gasdermin protein family. At present, there are six homologous genes in humans, including Gasdermin A (GSDMA), Gasdermin B (GSDMB), Gasdermin C (GSDMC), Gasdermin D (GSDMD), Gasdermin E (GSDME) and Pejvakin (PJVK). Pyroptosis mainly has two pathways, respectively the classical pathway relying on caspase-1 and the non-classical pathway relying on caspase-4/5/11. Gasdermin protein can be activated and cut through these two pathways, leading to the formation of pores on the cell membrane (Ding et al. 2016; Aglietti et al. 2016). Then, the balance of ion concentration inside and outside the cell is broken. After that, the cells swell, the plasma membrane is dissolved, and chromatin is fragmented while the pro-inflammatory components in the cells are released, resulting in cell death, that is, pyroptosis occurs.

In addition, recent studies found that caspase-3 can promote the occurrence of pyroptosis by activating the pyroptosis-related gene GSDME. At the same time, Shaofeng team found that serine protease GZMA can perform drilling function by hydrolyzing GSDMB in the first time, thus inducing pyroptosis of target cells. GSDMB is tissue-specific and highly expressed in epithelial-derived tumor cells of the digestive system. It can be seen that the induction of pyroptosis by GSDMB can enhance anti-tumor immunity which is expected to become a therapeutic target for digestive system tumors.

The relationship between scorched death and tumor is extremely complex. More and more studies have shown that scorched death affects the proliferation, invasion and metastasis of tumor cells, and then affects the prognosis of cancer (Liu et al. 2016; Sarhan et al. 2018). Studies have shown that caspase-1-mediated inflammation plays an important role in the pathogenesis of Barrett's esophagus and its progression to esophageal adenocarcinoma (Barber et al. 2020). In addition, the expression of pyroptosis related gene GSDME in esophageal squamous cell carcinoma was higher than that in normal cells, while the high expression of GSDME was associated with pyroptosis (Wu et al. 2019). However, the prognostic value of pyroptosis-related genes (PRGs) in ESCA has not been clarified.

In this study, we researched the expression profile of PRGs, its prognostic significance and related regulatory axis in ESCA through bioinformatics analysis, and to explore the relationship between PRGs and immune microenvironment in ESCA, and to predict the response of ESCA to immunotherapy. Our findings may provide new reference for prognostic biomarkers and therapeutic targets of ESCA, and may open up new possibilities for immunotherapy for ESCA.

## Materials and methods

### Datasets

Data of 185 ESCA patients obtained from The Cancer Genome Atlas (TCGA) (https://portal.gdc.cancer.gov/) were used as a training set. In addition, data of 179 patients was extracted from the Gene Expression Omnibus (GEO) (http://www.ncbi.nlm.nih.gov/geo/) dataset number GSE53625 as a validation set. At the same time, 394 PRGs were taken from the GeneCards website (https://www.genecards.org).

### Consensus clustering

The clustering was performed using ConsensusClusterPlus (Wilkerson and Hayes 2010), with resampling 80% of the samples for 10 repetitions and agglomerative pam clustering. Empirical cumulative distribution function plots were used to determine the optimal number of clusters.

### Identification of differentially expressed genes (DEGs)

With the help of the R software package *t* test function, each gene's significance was evaluated between the comparison group and control group. Furthermore, using *p* adjust, significant FDR is calculated for each gene, and finally, the difference information is calculated for each gene. As the screening criteria for mRNA differential expression, adjusted |log2 fold change|> 1.5 and *P* < 0.05 were applied.

### Functional enrichment analysis of gene set

In order to obtain the latest Kyoto Encyclopedia of Genes and Genomes (KEGG) Pathway gene annotation, we use the KEGG Rest API (https://www.kegg.jp/kegg/rest/keggapi.html), map the gene to the background set. Moreover, in order to obtain the result of gene set enrichment, use R software package clusterProfiler (version 3.14.3). We considered statistically significant a value of *P* < 0.05 and a FDR of < 0.1 in gene sets of 5 and 5000, respectively.

### Gene set enrichment analysis (GSEA)

Gene Set Enrichment Analysis (GSEA) (version 3.0) software was obtained from GSEA (http://software.broadinstitute.org/gsea/index.jsp). The datas were divided into two groups depending on PRGs low and high, and from Molecular Signatures Database (http://www.gsea-msigdb.org/gsea/downloads.jsp) downloaded the c2.cp.kegg.v7.4.symbols.gmt subcollection to determine the related pathways and molecular mechanisms, based on a phenotypic grouping and gene expression profile, in which, the minimum gene set is 5 and the maximum gene set is 5000, a thousand resampling times, *P* value of < 0.05 and a FDR of < 0.25 were considered statistically significant.

### Characterization of immune landscape between two prgs subgroups

An analysis of 184 ESCA samples was performed in SangerBox (vip.sangerbox.com/login.html) to determine the relative frequency of 20 immune cell types, and we observed the corresponding immune characteristics. A total of 184 samples were detected for mutation, of which the drawing sample contained 181 (98.4%). After that, the characteristics of 20 immune cell types were compared between the two PRGs subgroups, and the results were presented in a landscape map to illustrate and summarize the mutant genes.

### Survival analysis

We compared the overall survival time (OS) between PRGs high and low risk cohorts with the R package survival, through integrating survival time, survival status, and gene expression data for Kaplan–Meier (KM) analysis. A Cox method was used to evaluate the prognostic significance of each gene, and whether the risk score was an independent risk factor for OS in ESCA.

### Establishment and validation of risk model

A regression analysis was performed using Least absolute shrinkage and selection operator (LASSO) to reduce the prognostic genes previously previously filtered by “glmnet” R packet. In addition, tenfold cross verification was set up and the minimum lambda was defined as the optimal value to obtain the optimal model.

### Statistical analyses

R packet “rms” was used to integrat datas. Then, using cox method to establish the nomogram, and the prognostic significance of these features was analyzed in ESCA samples.

## Results

### Consensus clustering identified two PRGs subtypes

**Fig. 1:**
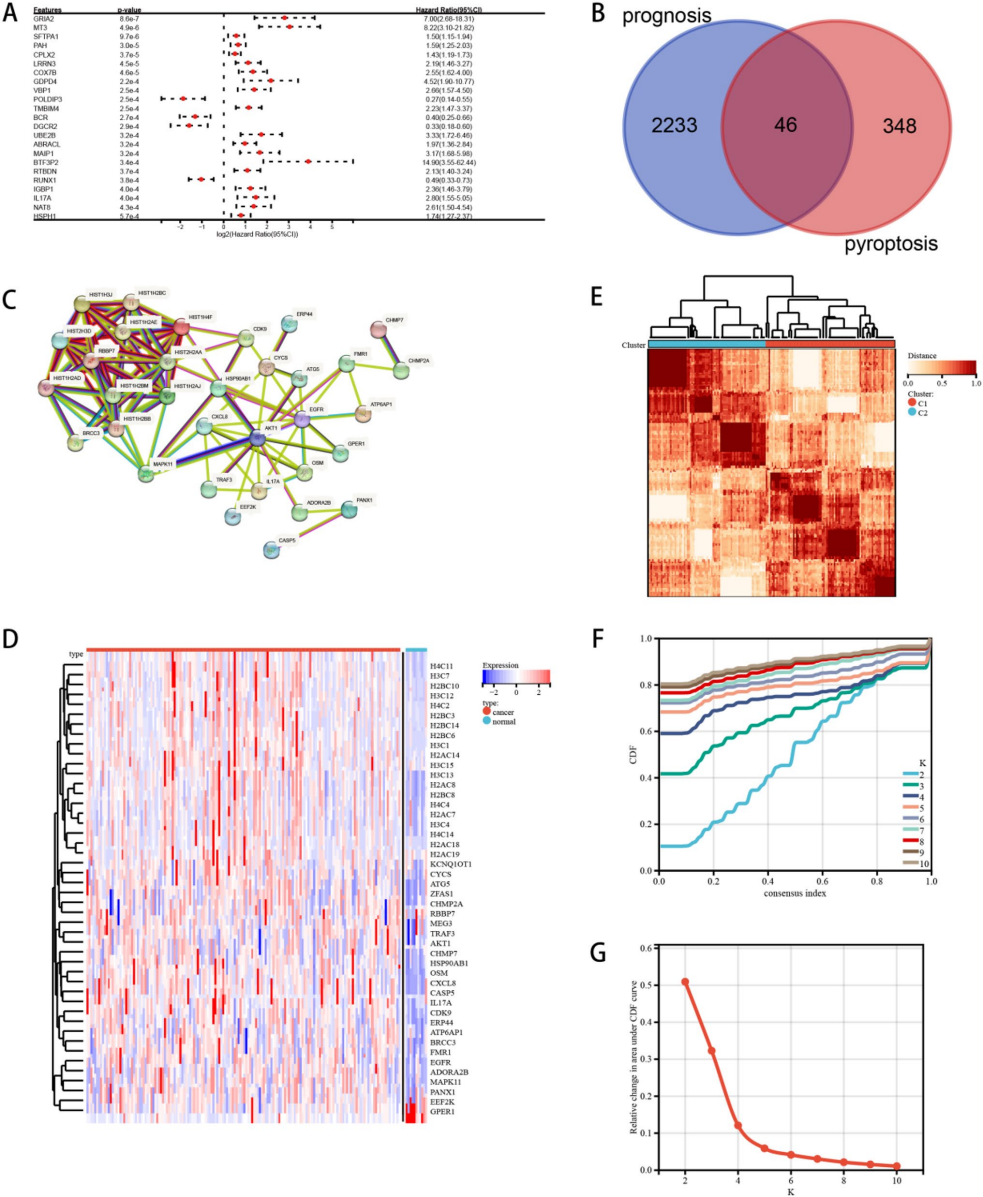
Consensus cluster. **a **Univariate Coxanalyse evaluates significant genes related to the prognosis of ESCA, **b **ESCA prognosis-related genes and PRGs take venn intersection, **c** protein–protein interactions among the PRGs, **d** The heatmap shows the expression of 46 PRGs in TCGA samples compared to normal samples; **e**consensus clustering heatmap of 46 genes in esophageal cancer samples, **f–g** Consensus clustering performed optimally with *K* = 2

First of all, 2279 significant genes related to the prognosis of ESCA (Fig. 1a) were obtained by univariate Cox analysis, and 46 PRGs (Fig. 1b) related to the prognosis of ESCA were obtained by venn intersection. Using STRING database to carry out protein–protein interaction (PPI) network analysis to further reveal the relationship between these PRGs (Fig. 1c). In addition, a comparison between normal and ESCA samples was also conducted to examine PRG expression patterns. Most of the PRGs are overexpressed in ESCA, such as H4C11 H3C7, ZFAS1, CYCS, CDK9, FMR1, HSP90AB1, OSM, CXCL8, and so on. (Fig. 1d).

Next, we use consensus clustering to determine the PRGs clustering of EC (Fig. 1e). Optimal cluster stability was determined when *K* = 2 (Fig. 1f–g). By the true no clustering method, the standard was divided into C1 and C2.

### Differentially expressed genes (DEGs) and signal pathways in high-pyroptosis subtype and low-pyroptosis subtype

**Fig. 2:**
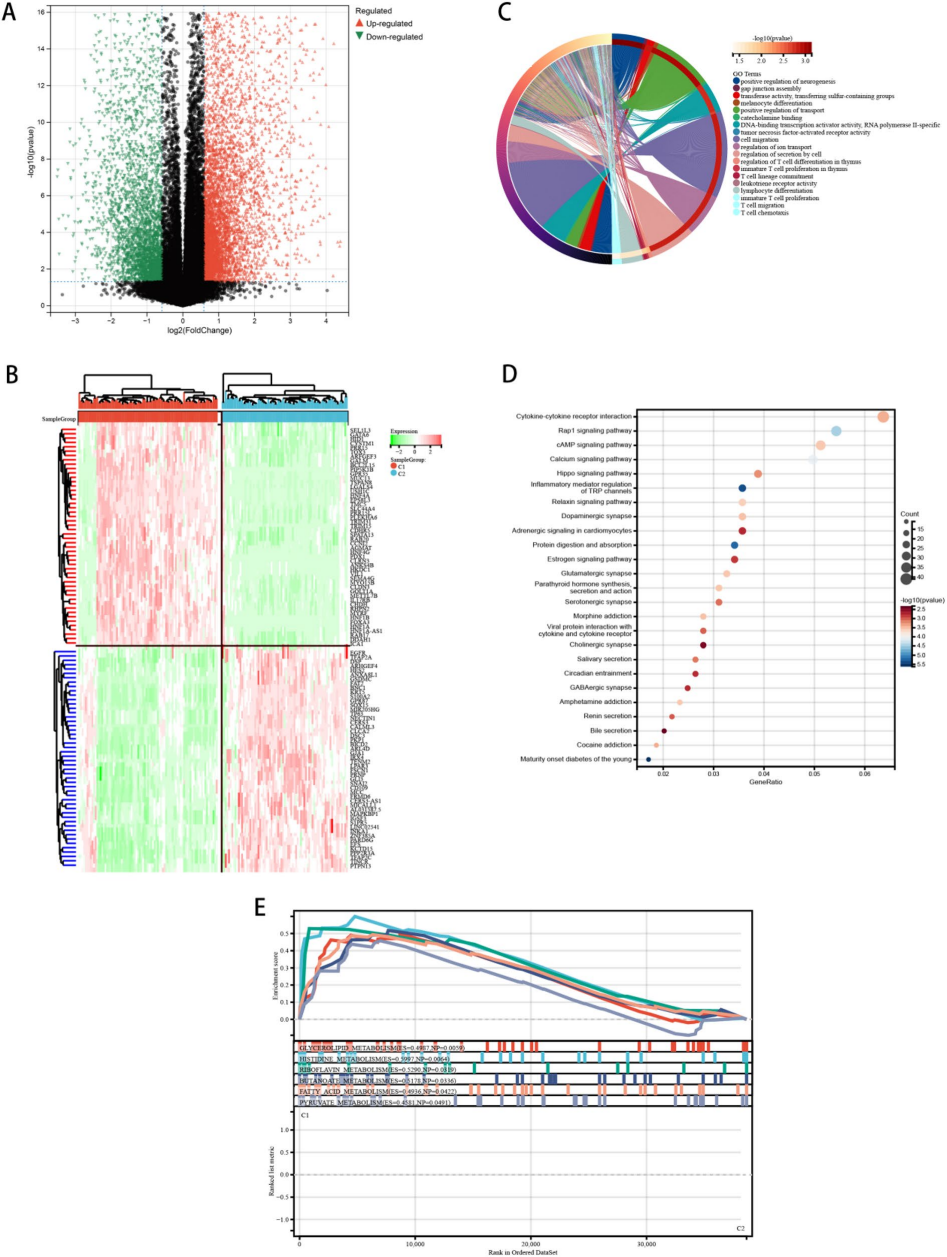
Different pyroptotic subtypes exhibit different differentially expressed genes (DEGs). **a** Volcano plot showing DEG distributions among PRG high and low subtypes in the TCGA cohort with a threshold of |log2 fold change|> 1.5 and *P* < 0.05, **b** heatmap showing the distribution of different PRGs subtypes DEGs expression,** c** circle diagram presents KEGG signaling pathway enrichment analysis, bubble diagram presents GO signaling pathway enrichment analysis, **d** GSEA analysis finds signaling pathways between PRGs high subtype and PRGs low subtype

For further analysis of the relationship between scorched death and esophageal cancer prognosis, a molecular mechanism by which high expression subtypes regulate prognosis was elucidated by identifying differential genes (DEGs) and signal pathways related to each subtype. Here, we found a collection of 3144 dysfunctional genes (Fig. 2a, b). In the subtype which PRGs are high, upregulated genes were enriched in immune-related pathways, including cytokine interaction, and so on. (Fig. 2c, d), which indicates that pyroptosis high subtypes are likely to be closely correlated with immune active microenvironments. To find out which signal pathways are activated in the high subgroup, we compared the GSEA between the high and low groups. The results show that the gene sets are rich in differences among different focal subtypes since they are closely related to a variety of metabolic pathways (Fig. 2e).

### Somatic mutation and tumor microenvironment landscape of different pyroptosis subtypes

**Fig. 3:**
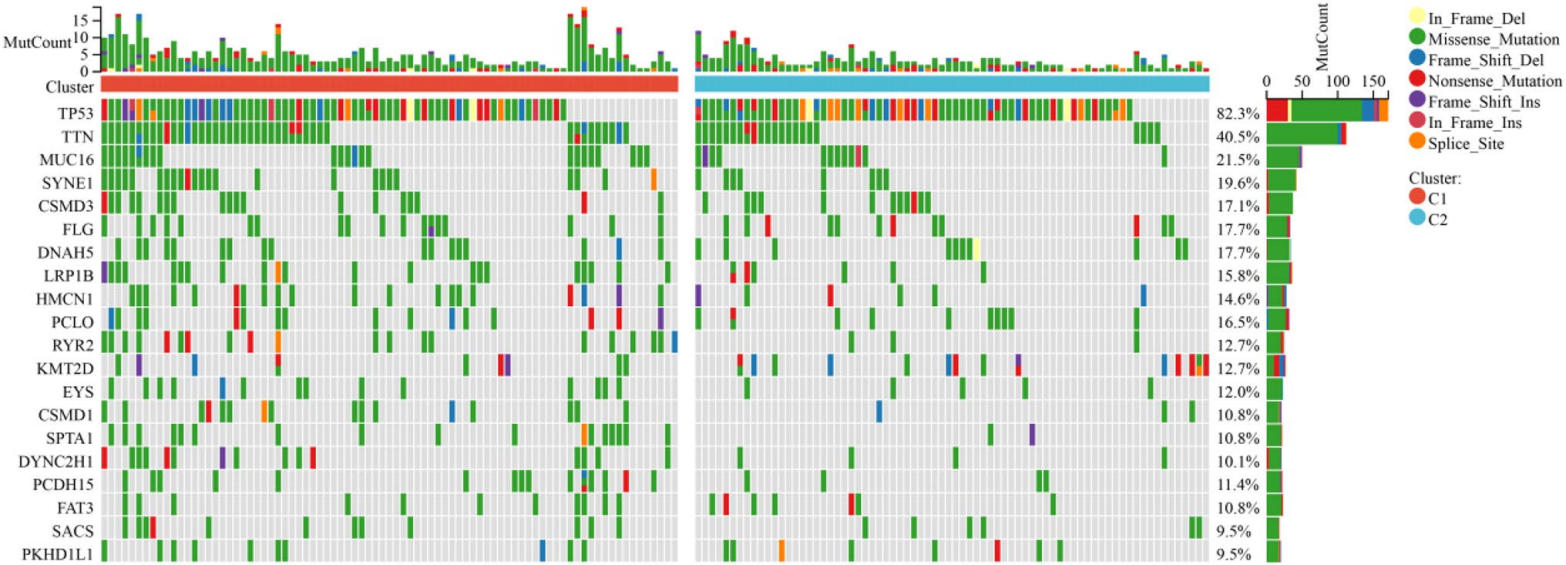
Comparison of somatic mutations among different PRGs subtypes

**Fig. 4:**
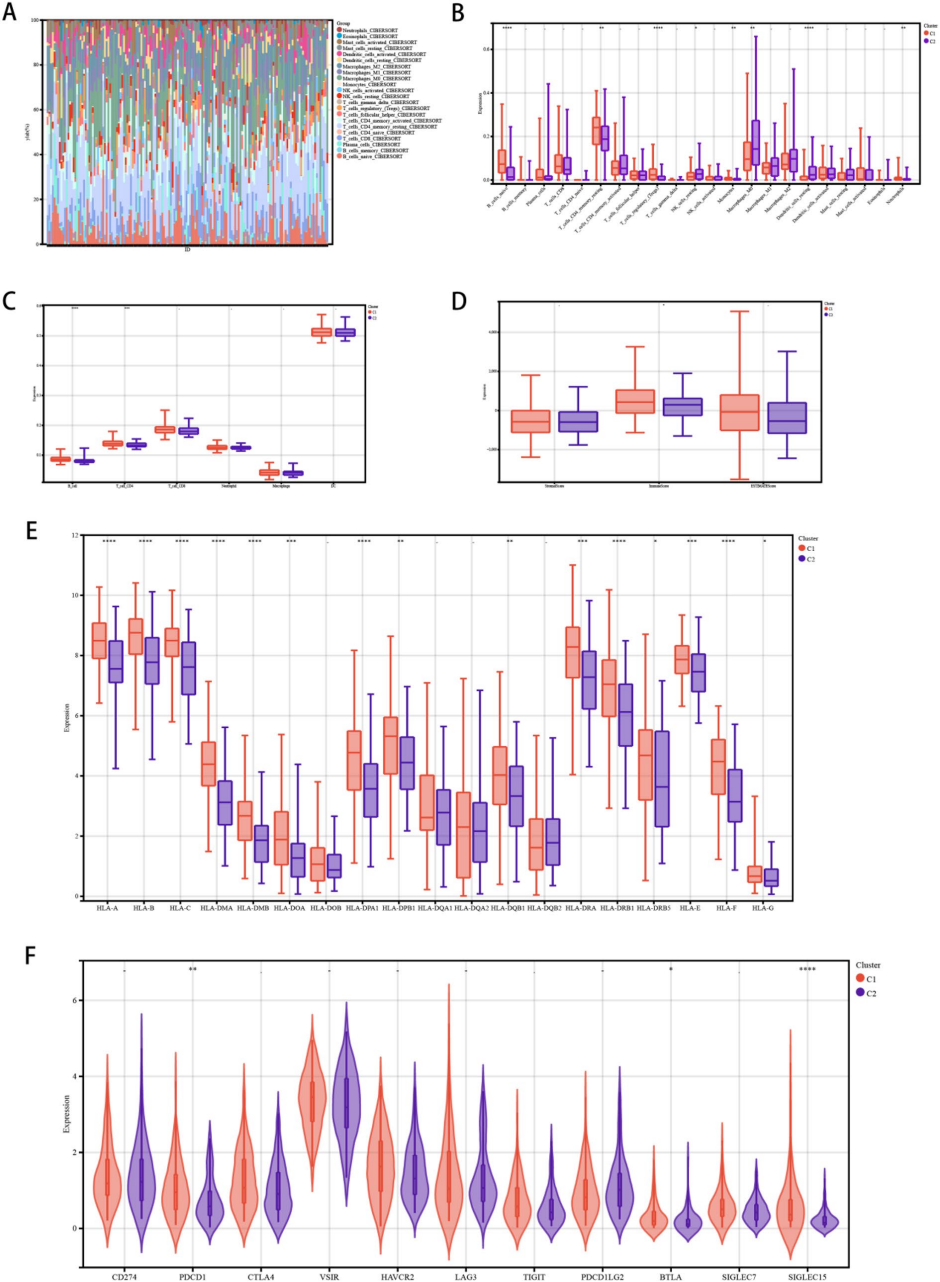
The immune landscape of PRGs-high among with PRGs-low subtypes. **a** Relative proportion of immune infiltrates of PRGs-high and PRGs-low subtypes, **b** boxplot visualization of immune cells significantly different between different subtypes, **c** violin plot visualization of six types of immune filtering cells assessed by TIMER Abundance, **d** boxplot visualization of ESTIMATE scores, **e** boxplot showing the differential expression of human leukocyte antigen genes among PRGs high subtypes and PRGs low subtypes, **f** violin plot showing multiple immune Differential expression of checkpoints among PRGs high and PRGs low subtypes.*P < 0.05, **P < 0.01, ***P < 0.001

We observed that there was an obvious somatic mutation spectrum between the high subtype and the low subtype (Fig. 3). High-frequency TP53 and TTN mutations are manifested in different subtypes, which were 82.3 and 40.5%, respectively. In addition, the mutations of MUC16 and SYNE1 were 21.5% and 19.6% respectively, which were relatively high. More and more evidence shows that pyroptosis affects the activation of some anti-tumor immune responses to a great extent. We assessed the difference in immune infiltration of 22 immune cells between the two subtypes through CIBERSORT. (Fig. 4a). The results which showed that the percentage of B cells naive, T cells CD4 memory resting, T cells regulatory (Tregs), Monocytes and Neutrophils increased significantly in patients with high scorching subtype (Fig. 4b). TIMER and ESTIMATE analysis also showed that high and low pyroptosis subtypes had a great influence on immune infiltration, in which the percentage of B cell and T cells CD4 in patients with high focus death subtype was significantly increased (Fig. 4c, d). In addition, most human leukocyte antigen genes have been observed to be significantly upregulated in the hyperpyrosis subtype and downregulated in the hypopyrosis subtype (Fig. 4e). And immune checkpoints PD-1 and SIGLEC15 were significantly up-regulated in the high-pyroptosis subtype (Fig. 4f). It is illustrated that the high-pyroptosis subtype is concerned with immunophenotype, and the low-pyroptosis subtype is related to immune cold phenotype.

### Construction and verification of pyroptosis risk model

**Fig. 5:**
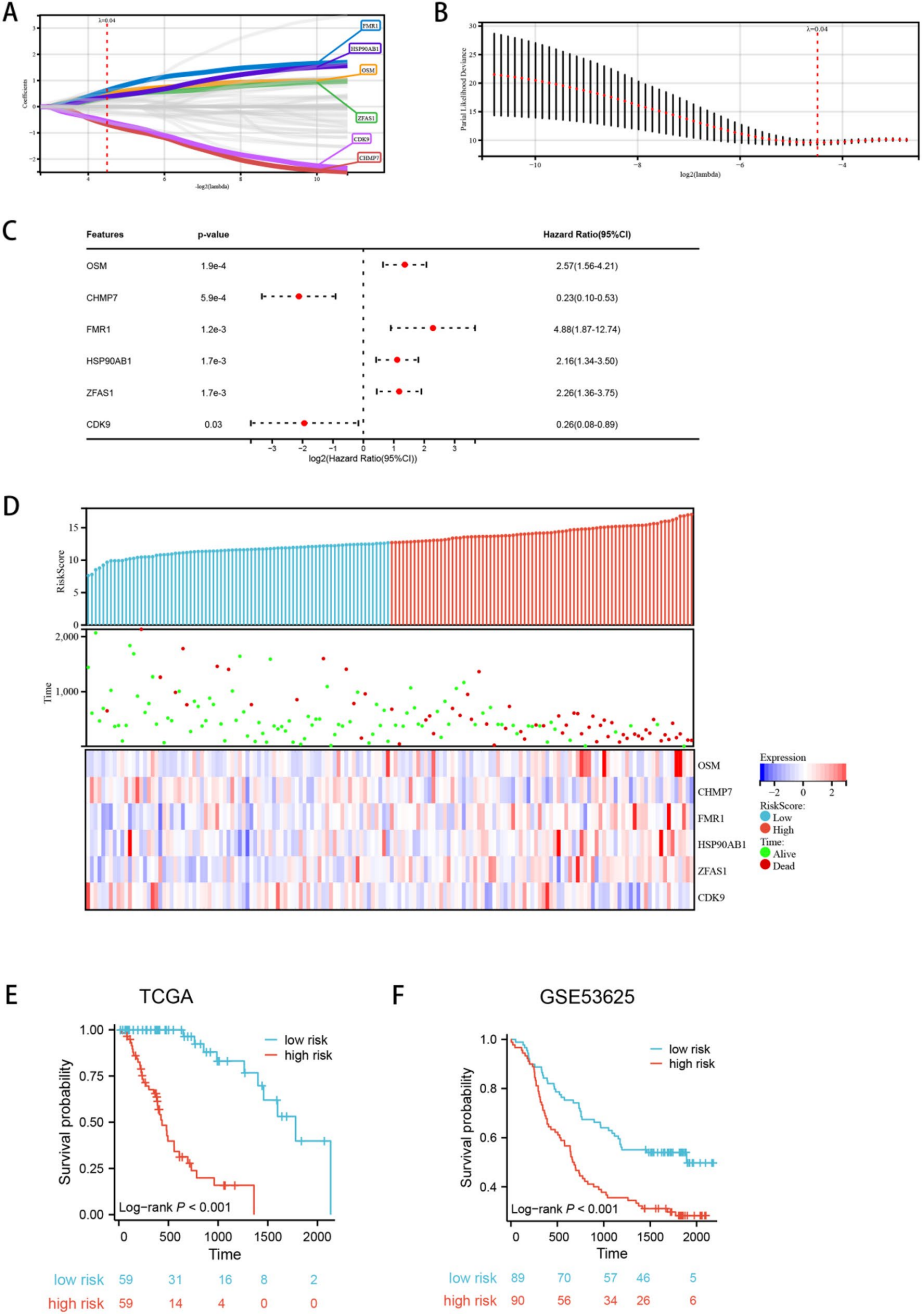
Construction and validation of PRGs risk models. **a–b** Lasso analysis identified the genes most associated with OS in the TCGA dataset, **c** univariate Cox analysis evaluated the prognostic value of PRGs genes in terms of OS, **d** heatmap of risk score, survival status and 6 prognostic gene signatures in TCGA database; Kaplan–Meier analysis in TCGA **e** and GSE53625 **f** cohort

On the basis of the above work, we established a prognostic model according to PRGs. In LASSO regression analysis, the PRGs were detected and streamlined and selected to build a predictive model (Fig. 5a, b). Subsequently, in COX univariate analysis, the results showed a significant correlation between different PRG subtypes and the patient’s OS (Fig. 5c). Then our further analysis of the relationship among survival status and risk score showed that the number of survival states in low-risk cohorts was remarkably higher than that in high-risk cohorts (Fig. 5d). Since then, the importance of this risk condition in ESCA has been further assessed in the TCGA queue using KM analysis (Fig. 5e). The results showed that the high-risk score corresponded to a poorer OS in ESCA, which was further confirmed by the results in the GEO queue (Fig. 5f).

### The independence of the risk model constructed

**Fig. 6:**
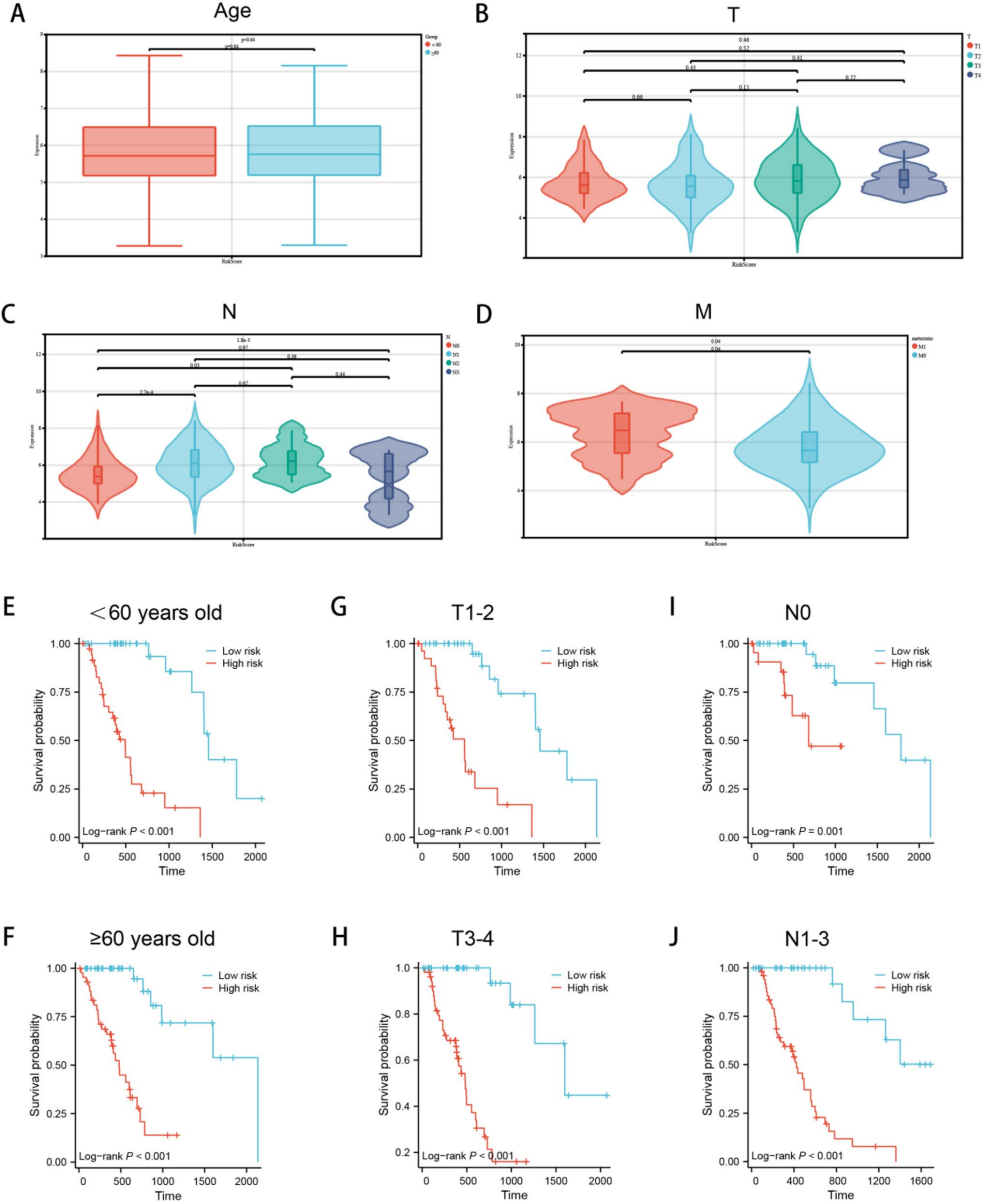
Correlation of risk score with clinical features. No significant differences were found among patients with different age **a**, T stage **b**, N stage **c**, and M stage **d,** independence Analysis of Risk Models, survival curves of patients regrouped according to age **e, f**, T stage **g, h** and N stage **i, j,** **k** multivariate Cox analysis

Next, we further analysed the relationship between risk scores and clinical characteristics, and evaluate the independence of the risk model through subgroup analysis and regression analysis. There was no significant difference in risk scores among patients with different age (Fig. 6a) and T stage (Fig. 6b), but there was significant difference between patients with different N stage (Fig. 6c) and M stage (Fig. 6d), indicating that the risk score was not associated with gender and T stage, but was closely related to N and M stages (Fig. 6a–d). In addition, when patients were re-grouped according to age (Fig. 6e, f), T stage (Fig. 6g, h) and N stage (Fig. 6i, j), the predictive power of the risk model is still very strong, and patients with lower risk scores have a significantly superior prognosis. This was followed by a multivariate Cox analysis (Fig. 6k), which also showed that the risk score of focal death can be an independent prognostic factor in ESCA.

### Construction and calibration of an integrated monogram

**Fig. 7:**
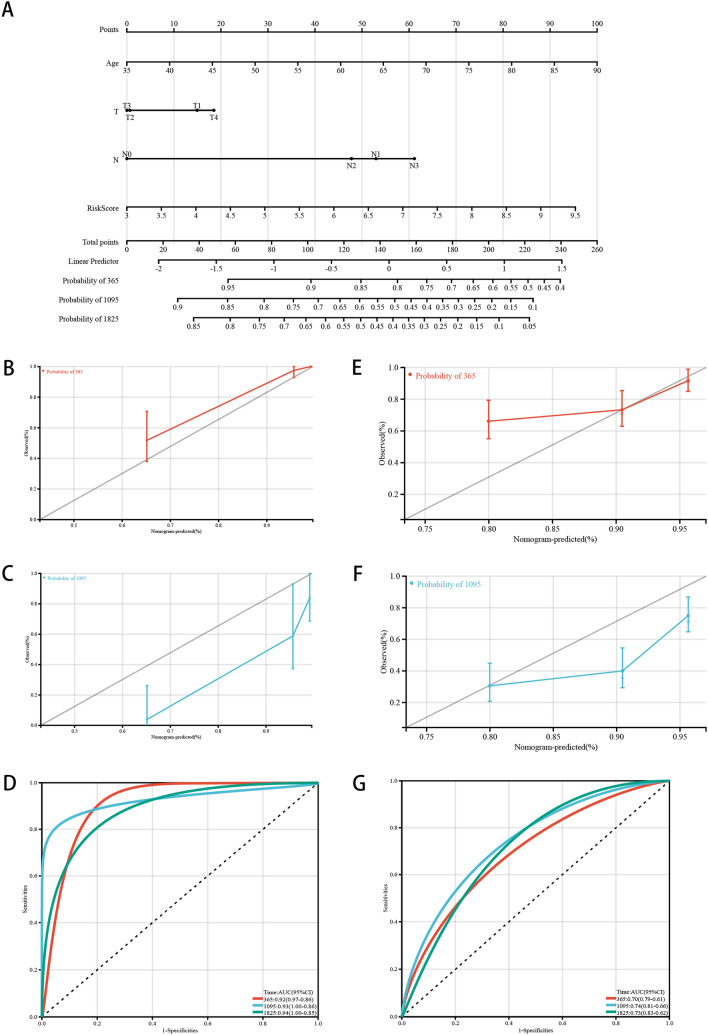
Construction of nomogram. **a** Nomogram, **b–d** Calibration curve and ROC analysis in the training cohort, **e–g** Calibration curve and ROC analysis in the validation cohort

Finally, to accurately predict the prognosis of patients with ESCA, a nomogram (Fig. 7a), which combines risk model with clinical characteristics, was constructed. A specific score was given according to the contribution of risk score and pathological features to the prognosis of ESCA. Then, based on the TCGA training queue and the GEO verification queue, we verified the nomogram. At the same time, the acceptable accuracy is shown in the model diagnosis of calibration curve (Fig. 7b, c, e, f) and ROC analysis (Fig. 7d, g). The overall survival observed in the training cohort preferably matched with the 3-year actual survival rate in the training cohort (Fig. 7a–d), and similar results were observed in the validation cohort (Fig. 7e–g). The results show that the integral nomogram can accurately predict the prognosis of patients with ESCA. It is further verified that the risk model can reliably and accurately predict the prognosis of patients with ESCA.

## Discussion

ESCA is a global malignant tumor, which has a high incidence and poor prognosis in East Asia. At present, TNM staging system is still used as the main factor to evaluate the prognosis of cancer patients. However, due to its limitations, its sensitivity and specificity in the evaluation of prognosis are poor. Therefore, it is particularly important to develop effective risk stratification methods and personalized targeted therapy strategies.

This work mainly focuses on the prognostic value of pyroptosis-related genetic features in patients with ESCA. In TGCA training set, we first identified the genes related to the prognosis of ESCA by Cox regression analysis, and intersected with the PRGs obtained in GeneCards, and obtained the PRGs related to the prognosis of ESCA. After that, the pyroptosis-related clusters of esophageal cancer were further determined by consensus clustering.

After the PRGs clustering was obtained, we identified the differential genes and signal pathways in each group, in order to understand the molecular mechanism of the high-pyroptosis subtype regulating prognosis, which was explained by GSEA analysis. The relationship among different PRG subtypes and immune microenvironment and immune checkpoint was analyzed. The results showed that the higher subtype of PRG was related to higher level of immune infiltration and higher expression of PD-1, which further indicated that the high expression of PRGs was associated with the activation of anti-tumor immune response, which partly clarified the potential mechanism.

Next, we establish a prediction model through Lasso regression analysis and Cox analysis, and use K–M analysis to further determine the importance of this risk situation. The GEO queue validates this conclusion as an authentication queue. In addition, we also evaluated the independence of the risk model by subgroup analysis, regression analysis and multivariate Cox analysis. The results show that the pyroptosis risk score can be used as an independent prognostic factor for patients with ESCA.

To further analyze the effect of pyroptosis on ESCA and evaluate the prognostic value of PRGs in ESCA patients, we constructed a prognostic risk model of PRGs and validated them in the validation cohort. In this study, six genes used to establish risk model. Oncostatin M (OSM) can promote the potential of breast cancer metastasis (Covert et al. 2019). Charged multivesicular body protein 7 (CHMP7) has been shown to be a risk gene for squamous cell carcinoma of the head and neck (Chen et al. 2022). Fragile X mental retardation 1 (FMR1) has been identified as a new immune-related prognostic biomarker for renal clear cell carcinoma (Wu et al. 2022). In addition, FMR1 can promote the development of colorectal cancer cells by stabilizing EGFR mRNA (Hu et al. 2022). Studies have shown that FMR1 is closely related to the prognosis of various tumors. Heat shock protein 90 alpha family class B member 1 (HSP90AB1) can promote the proliferation, migration and glycolysis of head and neck squamous cell carcinoma, and play an important role in epithelial mesenchymal transformation in the progression of gastric cancer (Zhang et al. 2022a, b; Wang et al. 2019). Zinc Finger NFX1-Type Containing 1 (ZFAS1) can forecast the clinical outcome of patients with different neoplasms including colorectal cancer, gastric cancer, and other types of cancer (Ghafouri-Fard et al. 2021). Cyclin-dependent kinase 9 (CDK9) can predict the prognosis of osteosarcoma, bladder cancer and other tumors, and can be used for targeted treatment of glioblastoma (Ma et al. 2019; Borowczak et al. 2022; Ranjan et al. 2021). Survival analysis shows that the risk model we established has a strong predictive performance which can predict the survival of patients with ESCA, whether in the training queue or the verification cohort. In addition, independence analysis and subgroup analysis showed that the established risk model could independently predict the prognosis of ESCA, regardless of age, sex and T, N stage. Finally, the line map of the integrated risk score also establishes and calibrates clinical features, which show considerable characteristics for predicting survival.

In recent years, tumor radiotherapy and chemotherapy are booming. In spite of this, the 5 year survival rate of ESCA is still not ideal (Zhang et al. 2022a, b). Therefore, it is particularly urgent to effectively classify patients according to their risk scores and carry out targeted and individualized treatment. Among them, risk stratification and targeted gene identification by bioinformatics analysis are feasible methods.

In ESCA, our study has its unique advantages. Our work focused on the characteristics of pyroptosis in patients with ESCA, and two molecular subpopulations were identified by consensus clustering, which prognosis and immune status varied significantly. On the basis of this, the potential biological mechanism was explored and partially clarified, and the effect of pyroptosis on the prognosis was clarified. At the same time, this work uses the TCGA data set as the training set and the GEO data set as the verification set. The samples are abundant and the results are more convincing, which provides excellent theoretical guidance for further research on ESCA. No one has studied the relationship between PRGs and ESCA before. Our study is a new attempt, which may provide a new idea for accurate prediction of the prognosis of ESCA. At the same time, it provides a new possibility of targeted therapy and immunotherapy for ESCA.

In addition, our research also has some limitations. On the one hand, all our results come from bioinformatics analysis, and further experiments are needed to verify our results. On the other hand, more prospective studies are needed to confirm the important value of PRGs in predicting the prognosis of ESCA.

In conclusion, our study emphasized the correlation between PRGs subtypes and the microenvironmental changes of ESCA immune tumors. These results may be helpful to the intervention based on immunotherapy in patients with ESCA. At the same time, we also constructed and verified the PRGs prognostic model, which is of great value in predicting the prognosis of patients with ESCA.

## Data Availability

The datasets generated during and/or analysed during the current study are available from the corresponding author on reasonable request.
